# Application of Phosphine-Phosphite Ligands in the Iridium Catalyzed Enantioselective Hydrogenation of 2-Methylquinoline

**DOI:** 10.3390/molecules15117732

**Published:** 2010-10-29

**Authors:** Miguel Rubio, Antonio Pizzano

**Affiliations:** Instituto de Investigaciones Químicas, Consejo Superior de Investigaciones Científicas and Universidad de Sevilla, Avda. Américo Vespucio 49, 41092 Sevilla, Spain

**Keywords:** asymmetric hydrogenation, chiral ligands, phosphines, phosphites, iridium, quinolines

## Abstract

The hydrogenation of 2-methylquinoline with Ir catalysts based on chiral phosphine-phosphites has been investigated. It has been observed that the reaction is very sensitive to the nature of the ligand. Optimization of the catalyst, allowed by the highly modular structure of these phosphine-phosphites, has improved the enantioselectivity of the reaction up to 73% *ee*. The influence of additives in this reaction has also been investigated. Contrary to the beneficial influence observed in related catalytic systems, iodine has a deleterious effect in the present case. Otherwise, aryl phosphoric acids produce a positive impact on catalyst activity without a decrease on enantioselectivity.

## 1. Introduction

The use of two coordinating functions of different nature in a chiral ligand constitutes as a very powerful approach in the field of asymmetric hydrogenation [1]. Thus, excellent catalyst performance has been achieved in a plethora of reactions by the use of ligands which appropriately combine diverse C, N, S and P donor groups [2,3,4,5].

**Figure 1:**
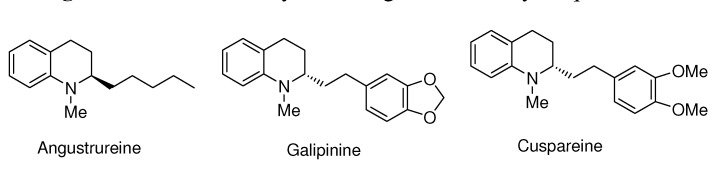
Some naturally occurring chiral tetrahydroquinolines.

The catalytic asymmetric hydrogenation of quinolines to produce optically active tetrahydro-quinolines is a very interesting reaction due to the importance of the resulting products. For instance, chiral tetrahydroquinolines are ubiquitous products in Nature (Figure 1) which exhibit, in addition, a wide range of biological properties of interest to the pharmaceutical industry [6,7,8,9,10,11,12]. A variety of catalytic systems, mostly based on Ir complexes and chiral chelating ligands with either equivalent(*C*_2_ symmetric) or non equivalent (*C*_1_ symmmetric) coordinating functions, have provided satisfactory results for this transformation. In this regard, Zhou *et al.* have described a catalytic system of [IrCl(cod)]_2_, MeO-Biphep and I_2_ that hydrogenates a variety of substituted quinolines with high enantioselectivities [13]. Likewise, Chan *et al.* have successfully applied other diphosphines such asP-phos and Difluorphos in this reaction [14,15]. Interestingly, this transformation can also be effectively catalyzed by species based on less donor diphosphonites, as shown by Reetz *et al.* [16]. Moreover, *C*_1_ symmetric phosphine-oxazoline, phosphine-sulfoximine or phosphine-phosphoramidite ligands have also led to efficient catalysts [17,18,19]. In addition, complexes based on a combination of a monodentate chiral phosphoramidite and an achiral phosphine, which provide good levels of activity and enantioselectivity, have been described by Feringa *et al.* [20]. Despite the excellent results obtained in these precedents, the knowledge about this reaction is still limited. Then, studies aimed to understand the influence of important features like ligand basicity, bite angle or the influence of additives are highly interesting [13,14,15,16,17,18,19,20,21].

**Figure 2:**
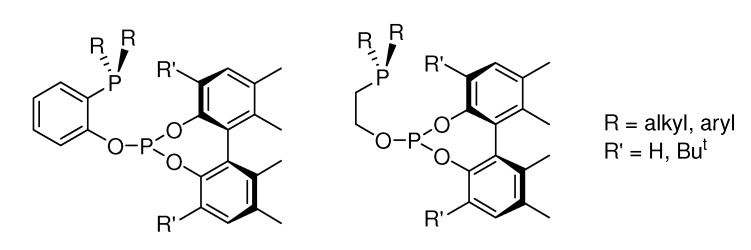
General structure of P-OP ligands.

Among chelating ligands with unequal coordinating groups, we have focused on phosphine-phosphites (P-OP) and their application in asymmetric catalytic hydrogenation reactions. Interestingly, the dissimilar electronic properties of their P functionalities provides an efficient differentiation between coordination positions, which reduces the number of reaction intermediates and allows a better stereocontrol [22,23]. Moreover, the highly modular structure of the P-OP derivatives developed in our laboratory (Figure 2), enables a detailed catalyst screening covering the influence of phosphine, phosphite and backbone fragments. This approach has successfully been applied in the Rh catalyzed enantioselective hydrogenation of several types of olefins [24,25,26]. In addition, we have demonstrated the usefulness of P-OP ligands in the Ir catalyzed hydrogenation of *N*-aryl imines [27]. As an extension of the scope of chiral phosphine-phosphites in the hydrogenation of C=N bonds, we describe herein preliminary results about the application of these ligands in the Ir catalyzed asymmetric hydrogenation of 2-methylquinoline.

## 2. Results and Discussion

**Figure 3:**
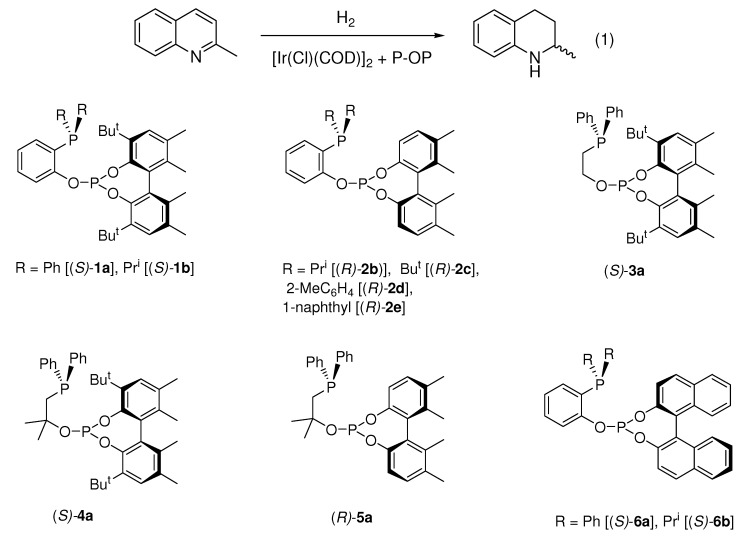
P-OP ligands used in the present study.

In a first stage, we have carried out a set of reactions to search for appropriate conditions for the catalytic hydrogenation of 2-methylquinoline (Eq 1). Thus, under 40 bar of hydrogen pressure and at room temperature, the catalyst generated from [Ir(Cl)(COD)]_2_ and ligand (*S*)-**1a** (Figure 3), produced only a moderate conversion and a low enantioselectivity (entry 1, Table 1). Interestingly, the presence of a more donating phosphine group in (*S*)-**1b** led to an important increase in catalyst activity, with a slight improvement on enantioselectivity (entry 2). Moreover, a cationic catalyst precursor based on (*S*)-**1b** generated a poorer catalyst (entry 3). Interestingly, the neutral catalysts produced an increased conversion at a lower pressure but again with low selectivities (entries 4, 5). From this preliminary screening, conditions of the latter reactions were chosen to analyze the influence of ligand structure on enantioselectivity (Table 2).

A comparison of the catalyst performance along the series of ligands allows one to extract some interesting observations. Most remarkably, the use of less hindered phosphite groups has a positive effect on the reaction. Thus, catalyst prepared with ligand (*R*)-**2b** produced an important increase on enantioselectivity over (*S*)-**1b**, from 16 to 62% *ee*, while maintaining a good conversion (entries 2, 3). Further examination of different phosphine groups did not allow us to improve the values achieved by(*R*)-**2b** (entries 4-6). As an alternative, we considered ligands based on ethane bridged examples, as this backbone has a positive effect in the hydrogenation of *N*-aryl imines [27]. Contrary to our expectations, the enantioselectivities with these ligands were rather low (entries 7-9). In an attempt to increase the practical utility of the present system we have also analyzed the performance of BINOL based ligands (*S*)-**6a** [28] and (*S*)-**6b**. These compounds are structurally similar to **2**, but considerably easier to synthesize, as the required chlorophosphite can be prepared in one step from commercially available BINOL [29]. New (*S*)-**6b** was readily prepared by condensation between 2-hydroxyphenyl-diisopropyl phosphine and BINOL chlorophosphite (see Experimental).

**Table 1:** Catalytic asymmetric hydrogenation of 2-methylquinoline with **P-OP** ligands.^1^

Entry	Cat. Precursor	P/atm	%Conv	% *ee*	Conf
1	½ [Ir(Cl)(COD)]_2_ * + (S)*-**1a**	40	48	7	*S*
2	½ [Ir(Cl)(COD)]_2_ * + (S)*-**1b**	40	84	27	*S*
3	[Ir(COD)(1b)]BF_4_	40	9	6	*n.d.*
4	½ [Ir(Cl)(COD)]_2_ * + (S)*-**1a**	20	72	0	*-*
5	½ [Ir(Cl)(COD)]_2_ * + (S)*-**1b**	20	96	16	*S*

^1^ Reactions were carried out at room temperature in toluene at a S/C = 100 and 0.6 M substrate concentration. Catalyst precursor was generated from [Ir(Cl)(COD)]_2_ and P-OP ligand at aIr:P-OP =1:1.1 ratio unless otherwise stated. Conversion was determined by ^1^H-NMR and enantiomeric excess (*ee*) by chiral HPLC. Configuration was determined by comparison of optical rotation to the literature value.

**Table 2:** Catalytic asymmetric hydrogenation of 2-methylquinoline with **P-OP** ligands.^1^

Entry	Ligand	P/atm	%Conv	% *ee*	Conf
1	*(S)*-**1a**	20	72	0	*-*
2	*(S)*-**1b**	20	96	16	*S*
3	*(R)*-**2b**	20	88	62	*S*
4^2^	*(R)*-**2c**	20	7	56	*S*
5^2^	*(R)*-**2d**	20	30	46	*S*
6	*(R)*-**2e**	20	34	20	*S*
7	*(S)*-**3a**	20	98	9	*S*
8	*(S)*-**4a**	20	100	0	*-*
9^2^	*(R)*-**5a**	20	6	10	*S*
10	*(S)*-**6a**	20	28	45	*R*
11	*(S)*-**6b**	20	82	65	*R*
12	*(S)*-**6b**	40	63	73	*R*
13	*(S)*-**6b**	10	40	63	*R*

^1^ Reactions were carried out at room temperature in toluene at a S/C = 100 and 0.6 M substrate concentration unless otherwise stated. Catalyst precursor was generated from [Ir(Cl)(COD)]_2_ and P-OP ligand at a Ir:P-OP =1:1.1 ratio. Conversion was determined by ^1^H-NMR and enantiomeric excess (*ee*) by chiral HPLC. Configuration was determined by comparison of optical rotation to the literature value. ^2^ 0.2 M substrate concentration.

Most remarkably, (*S*)-**6b** led to similar results as those obtained with (*R*)-**2b**. On the other hand,(*S*)-**6a** produced a moderate enantioselectivity, but lower than the ^i^Pr derivative. As shown by ligands **1**, a more electron donating ligand leads to a more active catalyst. Finally, examination of catalyst based on (*S*)-**6b** at different pressures exhibited an increase in enantioselectivity up to a 73% *ee* at 40 atm, but a lower value at 10 atm (entries 12, 13). Interestingly, product configuration depends on the nature of the phosphite fragment. Thus, the *S* product is favoured by ligands with a *tert*-butyl-substituted *S* phosphite group. On the contrary, ligands with an unsubstituted *S* phosphite fragment (e.g., (*S*)-**6a**) and (*S*)-**6b**), give predominantly the *R* amine.

As mentioned, the present hydrogenation is very sensitive to the presence of additives. In particular, excellent results have been reported in the literature by the use of iodine as cocatalyst [13,14,15,16,17]. Based on these precedents we prepared a set of reactions using different ligands in the presence of I_2_ (Table 3). As observed before, the cocatalyst produces an important increase in reactivity, leading to reaction completion in all cases. Noteworthily, the presence of iodine produces a reversal of product configuration in reactions performed with ligands **1**. However, the enantioselectivities were deceptively low in all cases. Presumably, coordination of π-acidic phosphite fragment in the Ir(III) species generated by iodine addition [30], should not be favoured, which may erode the chiral induction exerted by the P-OP ligand.

**Table 3:** Hydrogenation of 2-methylquinoline in presence of iodine.^1^

Entry	Ligand	P/atm	%Conv	% *ee*	Conf
1	*(S)*-**1a**	40	100	30	*R*
2	*(S)*-**1a**	20	100	16	*R*
3	*(S)*-**1b**	20	100	7	*R*
4	*(S)*-**3a**	20	100	0	*-*
5	*(S)*-**4a**	20	100	11	*R*
6	*(S)*-**6b**	40	100	0	*-*
7	*(S)*-**6b**	20	100	5	*R*

^1^ Reactions were carried out at room temperature in toluene at a S/C = 100 and 0.6 M substrate concentration. Catalyst precursor was generated from [Ir(Cl)(COD)]_2_, P-OP ligand and I_2_ at a Ir:P-OP:I_2_ =1:1.1:10 ratio. Conversion was determined by ^1^H NMR and enantiomeric excess (*ee*) by chiral HPLC. Configuration was determined by comparison of optical rotation to the literature value.

In order to improve the performance of catalyst based on (*S*)-**6b**, we have next studied the influence of other additives mentioned in the literature (Table 4). Several salts were tested first, although they did not provide a beneficial effect over the reference system (entries 1-5). On the contrary, an interesting effect is provided by phosphoric acids [31,32,33]. Thus, diphenylphosphoric acid produced a more active catalyst, although less enantioselective (entry 6). From this result and the reported asymmetric reduction of quinolines catalyzed by binaphthol based phosphoric acids [34], we have tested the two enantiomers of 1,1’-binaphthyl-2,2’-diylphosphoric acid (BINOL-PO_2_H, entries 7, 8). Interestingly, these acids also have a beneficial effect on catalyst reactivity and maintain the enantioselectivity. However, they did not show any influence of the configuration of the acid on enantioselectivity. Probably, more elaborated binaphthyl fragments, including aromatic groups in3,3’ positions, are needed to provide a synergistic effect to improve the catalytic system [35].

**Table 4:** Influence of diverse additives in the hydrogenation of 2-methylquinoline with ligand (*S*)-**6b**.^1^

Entry	Additive	P/atm	%Conv	% *ee*	Conf
1	none	20	82	65	*R*
2	piperidine·HCl	20	19	66	*R*
3	Bu_4_NI	20	63	62	*R*
4	KCl	20	80	36	*R*
5	NaBF_4_	20	69	49	*R*
6	(PhO)_2_PO_2_H	20	92	49	*R*
7	(*R*)-BINOL-PO_2_H	20	89	70	*R*
8	(*S*)-BINOL-PO_2_H	20	92	67	*R*

^1^ Reactions were carried out at room temperature in toluene at a S/C = 100 and 0.6 M substrate concentration. Catalyst precursor was generated from [Ir(Cl)(COD)]_2_, ligand **6b** and additive at a Ir:**6b**:additive =1:1.1:10 ratio. Conversion was determined by ^1^H-NMR and enantiomeric excess (*ee*) by chiral HPLC. Configuration was determined by comparison of optical rotation to the literature value.

## 3. Experimental

### 3.1. General

All reactions and manipulations were performed under nitrogen or argon, either in a Braun Labmaster 100 glovebox or using standard Schlenk-type techniques. All solvents were distilled under nitrogen using the following dessicants: Sodium-benzophenone-ketyl for benzene, diethylether (Et_2_O) and tetrahydrofuran (THF); sodium for petroleum ether and toluene; CaH_2_ for dichloromethane (CH_2_Cl_2_) and NaOMe for methanol (MeOH). NMR spectra were obtained on Bruker DPX-300, DRX-400 or DRX-500 spectrometers. ^31^P{^1^H} NMR shifts were referenced to external 85% H_3_PO_4_, while ^13^C{^1^H} and ^1^H shifts were referenced to the residual signals of deuterated solvents. All data are reported in ppm downfield from Me_4_Si. HPLC analyses were performed by using a Waters 2690 System. HRMS data were obtained using a Jeol JMS-SX 102A mass spectrometer. Optical rotations were measured on a Perkin-Elmer Model 341 polarimeter.

*(S)-2-(Diisopropylphosphino)phenyl-1,1’-binaphthyl-2,2’-diyl phosphite* [**(*S*)-6b**]. A solution of(*S*)-2,2’-bisnaphtoxyphosphorus chloride (0.35 g, 1.0 mmol) in toluene (10 mL) was added dropwise to (2-hydroxyphenyl)diisopropyl phosphine (0.21 g, 1.0 mmol) and NEt_3_ (0.15 mL, 1.1 mmol) dissolved in toluene (10 mL). The resulting suspension was stirred for 24 h, the mixture filtered and volatiles removed. The solid obtained was dissolved in toluene and passed through a short pad of neutral alumina. Solution was evaporated yielding a white solid (0.25 g, 50%). [α]^D^_20_ = +222 (c 0.5, THF). ^1^H- NMR (CDCl_3_, 500 MHz): δ 0.93 (m, 6H, 2 CH_3_, ^i^Pr), 1.08 (dd, *J*_HP_ = 14 Hz, *J*_HH_ = 7 Hz, 3H, CH_3_, ^i^Pr), 1.12 (dd, *J*_HP_ = 14 Hz, *J*_HH_ = 7 Hz, 3H, CH_3_, ^i^Pr), 2.18 (m, 2H, 2 CH, ^i^Pr), 7.12-7.22 (m, 2H, 2 H arom), 7.25-7.35 (m, 3H, 3 H arom), 7.37-7.50 (m, 5H, 5 H arom), 7.57 (d, *J*_HH_ = 8.3 Hz, 1H, H arom), 7.60 (d, *J*_HH_ = 8 Hz, 1H, H arom), 7.87-7.95 (m, 3H, 3 H arom), 8.00 (d, *J*_HH_ = 8.6 Hz, 1H, H arom). ^31^P{^1^H} NMR (CDCl_3_, 202.4 MHz): δ -2.3 (br, P-C), 143.1 (d, P-O, *J*_PP_ = 30 Hz); ^13^C{^1^H} NMR (CDCl_3_, 125.8 MHz): δ 19.5 (d, *J*_CP_ = 10 Hz, Me, ^i^Pr), 19.6 (d, *J*_CP_ = 10 Hz, Me, ^i^Pr), 20.0 (Me, iPr), 20.2 (Me, iPr), 23.2 (d, *J*_CP_ = 13 Hz, CH, ^i^Pr), 23.3 (d, *J*_CP_ = 13 Hz, CH, ^i^Pr), 120.1 (d, *J*_CP_ = 11 Hz, CH arom), 121.9 (2 CH arom), 122.9 (C_q_ arom), 123.8 (CH arom), 124.4 (d, *J*_CP_ = 5 Hz, C_q_ arom), 124.9 (CH arom), 125.1 (CH arom), 126.1 (CH arom), 126.3 (CH arom), 127.0 (CH arom), 127.1 (CH arom), 127.6 (d, *J*_CP_ = 23 Hz, C_q_ arom), 128.2 (CH arom), 128.3 (CH arom), 129.7 (CH arom), 130.1 (CH arom), 130.3 (CH arom), 131.2 (C_q_ arom), 131.6 (C_q_ arom), 132.6 (C_q_ arom), 132.9 (C_q_ arom), 135.0 (d, *J*_CP_ = 7 Hz, CH arom), 147.2 (C_q_ arom), 148.0 (d, *J*_CP_ = 4 Hz, C_q_ arom), 155.7 (dd, *J*_CP_ = 14, 6 Hz, C_q_ arom); HRMS (FAB): *m/z* 525, 1766, [M+H]^+^ (exact mass calculated for C_32_H_31_O_3_P_2_: 525.1748).

### 3.2. General Hydrogenation Procedure

In a glovebox, to a 2 mL glass vial was added 2-methylquinoline (0.3 mmol), the appropriate phosphine-phosphite ligand (3.15 μmol), [IrCl(COD)]_2_ (1.5 μmol) and the additive (30 μmol) in toluene (0.5 mL). Vials were placed in a model HEL CAT18 pressure reactor that holds up to eighteen reactions. The reactor was purged three times with H_2_ and finally pressurized. After 24 h, the reactor was slowly depressurized, solutions were evaporated and conversions were determined by ^1^H-NMR. The resulting mixtures were dissolved in a 95:5 *n*-hexane/isopropanol mixture and filtered through a short pad of silica to remove the catalyst. Enantiomeric excesses of 2-methyl-1,2,3,4-tetrahydroquinoline were analyzed by chiral HPLC (Chiracel OJ-H, flow 0.5 mL/min, *n*-hexane:isopropanol 95:5).

## 4. Conclusions

We have reported a preliminary study about the Ir catalyzed hydrogenation of 2-methylquinoline with phosphine-phosphite ligands **1**-**6**. The screening indicates an important influence of the ligand structure and has led to a convenient catalytic additive-free system which achieves a good conversion and an enantioselectivity of up to 73% *ee*. Complementary studies on the influence of additives indicates a deletorious effect of iodine. On the contrary, phosphoric acids have a positive influence on catalyst reactivity, without affecting enantioselectivity in the case of binaphthyl phosphoric acids. Studies to deep into these observations for the improvement of this catalytic system are currently under investigation.

## References

[1] Tang W., Zhang X. (2003). New chiral phosphorus ligands for asymmetric hydrogenation. Chem. Rev..

[2] Bell S., Wustenberg B., Kaiser S., Menges F., Netscher T., Pfaltz A. (2006). Asymmetric hydrogenation of unfunctionalized, purely alkyl-substituted olefins. Science.

[3] Cui X.H., Ogle J.W., Burgess K. (2005). Stereoselective hydrogenations of aryl-substituted dienes. Chem. Comm..

[4] Hoge G., Wu H.-P., Kissel W.S., Pflum D.A., Greene D.J., Bao J. (2004). Highly selective asymmetric hydrogenation using a three hindered quadrant bisphosphine rhodium catalyst. J. Am. Chem. Soc..

[5] Evans D.A., Campos K.R., Tedrow J.S., Michael F.E., Cagne M.R. (2003). Application of chiral mixed phosphorus/sulfur ligands to enantioselective rhodium-catalyzed dehydroamino acid hydrogenation and ketone hydrosilylation processes. J. Am. Chem. Soc..

[6] Keay J.D., Trost B.M., Fleming I. (1991). Comprehensive Organic Synthesis.

[7] Wang D.-W., Zhou Y.-G., Chen Q.-A., Wang D.-S., Nugent T.C. (2010). Chiral Amine Synthesis: Methods, Developments and Applications.

[8] Barton D.H.R., Nakanishi K., Meth-Cohn O. (1999). Comprehensive Natural Products Chemistry.

[9] Jacquemond-Collet I., Hannedouche S., Fabre N., Fouraste I., Moulis C. (1999). Two tetrahydroquinoline alkaloids from Galipea officinalis. Phytochemistry.

[10] Rokotoson J.H., Fabre N., Jacquemond-Collet I., Hannedouche S., Fabre N., Fouraste I., Moulis C. (1998). Alkaloids from *Galipea officinalis*. Planta Med..

[11] Houghton P.J., Woldemariam T.Z., Watanabe Y., Yates M. (1999). Activity against *Mycobacterium tuberculosis* of alkaloid constituents of angostura black, *Galipea officinalis*. Planta Med..

[12] Jacquemond-Collet I., Bessiere J.M., Hannedouche S., Bertrand C., Fouraste I., Moulis C. (2001). Identification of the alkaloids of Galipea officinalis by gas chromatography-mass spectrometry. Phytochem. Anal..

[13] Zhou Y.-G. (2007). Asymmetric hydrogenation of heteroaromatic compounds. Acc. Chem. Res..

[14] Tang W.-J, Tan J, Xu L.-J, Lam K.-H., Fan Q.-H., Chan A.S.C. (2010). Highly enantioselective hydrogenation of quinoline and pyridine derivatives with iridium-(P-Phos) catalyst. Adv. Synth. Catal..

[15] Tang W., Sun Y., Xu L., Wang T., Fan Q., Lam K.-H., Chan A.S.C. (2010). Highly efficient and enantioselective hydrogenation of quinolines and pyridines with Ir-Difluorphos catalyst. Org. Biomol. Chem..

[16] Reetz M.T., Li X. (2006). Asymmetric hydrogenation of quinolines catalyzed by iridium complexes of BINOL-derived diphosphinites. Chem. Commun..

[17] Lu S.-M., Han X.-W., Zhou Y.-G. (2004). Asymmetric hydrogenation of quinolines catalyzed by iridium with chiral ferrocenyloxazoline derived N,P ligands. Adv. Synth. Catal..

[18] Lu S.-M., Bolm C. (2008). Synthesis of sulfoximine-derived P,N ligands and their applications in asymmetric quinoline hydrogenations. Adv. Synth. Catal..

[19] Eggenstein M., Thomas A., Theuerkauf J., Franciò G., Leitner W. (2009). Highly efficient and versatile phosphine-phosphoramidite ligands for asymmetric hydrogenation. Adv. Synth. Catal..

[20] Mršić N., Lefort L., Boogers J.A.F., Minnaard A.J., Feringa B.L., de Vries J.G. (2008). Asymmetric hydrogenation of quinolines catalyzed by iridium complexes of monodentate BINOL-derived phosphoramidites. Adv. Synth. Catal..

[21] Lam K.H., Xu L., Feng L., Fan Q.-H., Lam F.L., Lo W.-H., Chan A.S.C. (2005). Highly enantioselective iridium-catalyzed hydrogenation of quinoline derivatives using chiral phosphinite H8-BINAPO. Adv. Synth. Catal..

[22] Suárez A., Méndez-Rojas M.A., Pizzano A. (2002). Electronic differences between coordinating functionalities of chiral phosphine-phosphites and effects in catalytic enantioselective hydrogenation. Organometallics.

[23] Suárez A., Pizzano A. (2001). New chiral phosphine-phosphites: a convenient synthesis based on the demethylation of *o*-anisyl phosphines and application in highly enantioselective catalytic hydrogenations. Tetrahedron: Asymm..

[24] Rubio M., Suárez A., Álvarez E., Pizzano A. (2005). Highly enantioselective hydrogenation of enol ester phosphonates catalyzed by rhodium phosphine-phosphite complexes. Chem. Commun..

[25] Rubio M., Vargas S., Suárez A., Álvarez E., Pizzano A. (2007). Tuning of the structures of chiral phosphane-phosphites: application to the highly enantioselective synthesis of *α*-acyloxy phosphonates by catalytic hydrogenation. Chem. Eur. J..

[26] Vargas S., Suárez A., Álvarez E., Pizzano A. (2008). Highly enantioselective hydrogenation of enol ester phosphonates: a versatile procedure for the preparation of chiral *β*-hydroxyphosphonates. Chem. Eur. J..

[27] Vargas S., Rubio M., Suárez A., del Río D., Álvarez E., Pizzano A. (2006). Iridium complexes with phosphine-phosphite ligands. Structural aspects and application in the catalytic asymmetric hydrogenation of *N*-aryl imines. Organometallics.

[28] Baker M.J., Pringle P.G. (1993). A tetraphos ligand with *C*_3_ symmetry. Chem. Commun..

[29] Nozaki K., Sakai N., Nanno T., Higashijima T., Mano S., Horiuchi T., Takaya H. (1997). Highly enantioselective hydroformylation of olefins catalyzed by rhodium(I) complexes of new chiral phosphine-phosphite ligands. J. Am. Chem. Soc..

[30] Wang D.-W., Wang X.-B., Wang D.-S., Lu S.-M., Zhou Y.-G., Li Y.-X. (2009). Highly enantioselective iridium-catalyzed hydrogenation of 2-belzylquinolines and 2-functionalyzed and 2,3-disubstituted quinolines. J. Org. Chem..

[31] Akiyama T. (2007). Stronger Brønsted acids. Chem. Rev..

[32] Tadaoka H., Cartigny D., Nagano T., Gosavi T., Ayad T., Genêt J.-P., Ohshima T., Ratovelomanana-Vidal V., Mashima K. (2009). Unprecedented halide dependence on catalytic asymmetric hydrogenation of 2-aryl and 2-alkyl-substituted quinolinium salts by using Ir complexes with Difluorphos and halide ligands. Chem. Eur. J..

[33] Wang D.-S., Zhou Y.-G. (2010). Asymmetric hydrogenation of quinolines activated by Brønsted acids. Tetrahrdron Lett..

[34] Rueping M., Antonchick A.P., Theissmann T. (2006). A Highly enantioselective Brønsted acid catalyzed Cascade reaction: organocatalytic transfer hydrogenation of quinolines and their application in the synthesis of alkaloids. Angew. Chem. Int. Ed. Engl..

[35] Li C., Wang C., Villa-Marcos B., Xiao J. (2008). Chiral counteranion-aided asymmetric hydrogenation of acyclic imines. J. Am. Chem. Soc..

